# Epidemiological Dynamics of SARS-CoV-2 Variants During Social Protests in Cali, Colombia

**DOI:** 10.3389/fmed.2022.863911

**Published:** 2022-03-31

**Authors:** Luz H. Patiño, Sergio Castañeda, Marina Muñoz, Nathalia Ballesteros, Angie L. Ramirez, Nicolas Luna, Enzo Guerrero-Araya, Julie Pérez, Camilo A. Correa-Cárdenas, Maria Clara Duque, Claudia Méndez, Carolina Oliveros, Maryia V. Shaban, Alberto E. Paniz-Mondolfi, Juan David Ramírez

**Affiliations:** ^1^Centro de Investigaciones en Microbiología y Biotecnología-UR (CIMBIUR), Facultad de Ciencias Naturales, Universidad del Rosario, Bogotá, Colombia; ^2^Agencia Nacional de Investigación y Desarrollo (ANID)—Millennium Science Initiative Program—Millennium Nucleus in the Biology of the Intestinal Microbiota, Santiago, Chile; ^3^Microbiota-Host Interactions and Clostridia Research Group, Facultad de Ciencias de la Vida, Universidad Andrés Bello, Santiago, Chile; ^4^Grupo de Investigación en Enfermedades Tropicales del Ejército (GINETEJ), Laboratorio de Referencia e Investigación, Dirección de Sanidad Ejército, Bogotá, Colombia; ^5^Incubadora Venezolana de la Ciencia (IVC), Centro de Investigaciones Biomédicas IDB, Barquisimeto, Venezuela; ^6^Molecular Microbiology Laboratory, Department of Pathology, Molecular and Cell-Based Medicine, Icahn School of Medicine at Mount Sinai, New York, NY, United States

**Keywords:** SARS-CoV-2, COVID-19, effective reproduction number, lineages, Cali

## Abstract

**Background:**

The third wave of the global health crisis attributed to the severe acute respiratory syndrome coronavirus 2 (SARS-CoV-2) virus reached Colombia in March 2021. Over the following 6 months, it was interpolated by manifestations of popular disapproval to the actual political regime—with multiple protests sprouting throughout the country. Large social gatherings seeded novel coronavirus disease 2019 (COVID-19) variants in big cities and propagated their facile spread, leading to increased rates of hospitalizations and deaths.

**Methods:**

In this article, we evaluate the effective reproduction number (Rt) dynamics of SARS-CoV-2 in Cali, Colombia, between 4 April 2021 and 31 July 2021 based on the analysis of 228 genomes.

**Results:**

Our results showed clear contrast in Rt values between the period of frequent protests (Rt > 1), and the preceding and following months (Rt < 1). Genomic analyses revealed 16 circulating SARS-CoV-2 lineages during the initial period—including variants of concern (VOCs) (Alpha, Gamma, and Delta) and variants of interest (VOIs) (Lambda and Mu). Furthermore, we noticed the Mu variant dominating the COVID-19 distribution schema as the months progressed. We identified four principal clusters through phylogenomic analyses—each one of potentially independent introduction to the city. Two of these were associated with the Mu variant, one associated with the Gamma variant, and one with the Lambda variant.

**Conclusion:**

Our results chronicle the impact of large group assemblies on the epidemiology of COVID-19 during this intersection of political turmoil and sanitary crisis in Cali, Colombia. We emphasize upon the effects of limited biosecurity strategies (which had characterized this time period), on the spread of highly virulent strains throughout Cali and greater Colombia.

## Introduction

In December 2019, the identification of an amply transmissible and highly virulent member of the *Coronaviridae* family (coronavirus disease 2019, COVID-19) in Wuhan, China informed the world of a novel pathogen which would seed an ensuing pandemic (1). As of 10 December 2021, laboratory diagnoses have confirmed 269,021,697 cases and more than 5 million deaths proceeding from COVID-19 universally.^1^ This statistic is dominated by the American continent, with Colombia being one of the countries most impacted by the spread of severe acute respiratory syndrome coronavirus 2 (SARS-CoV-2): 5,089,695 confirmed cases and 129,011 deaths.^2^

Colombia has faced three separate “waves” throughout the COVID-19 epidemic (May–September 2020; December 2020–February 2021; and March–August 2021). These delineations are correlated with the restraining and relaxing of social distancing measures implemented by the government in efforts to maintain balance between the health care burden and the precarious economic situation experienced by public and private enterprises (2). In the midst of the third wave (March--August 2021), a series of demonstrations took place throughout the country. Between 28 April 2021 and 31 July 2021, Colombians actively protested a new taxation proposal, inadequate federal management of the pandemic and ensuing poverty and unemployment seen throughout the country. At the moment of these protests, vaccination levels remained still low (with only 12,179,103 people, or 23.9% of the population having received both doses) and the clear infringement upon advisable social distancing protocol set off alarm in the public health sphere, as more people took to the streets disrespecting standards of biosecurity.^3^

The three largest cities in Colombia: Bogotá, Medellín, and Cali, then witnessed increase in the weekly averages of cases and deaths by SARS-CoV-2, as well as a concerning presence of novel viral lineages: Alpha (B.1.1.7), Gamma (P.1), Delta (B.1.617.2), and Mu (B.1.621) (see text footnote 2) (3).

Around the world, neither the onset of the pandemic, nor stringent lockdown protocols ever truly arrested the demonstration of public disagreement with modern social structures. From marches for the *Black Lives Matter* movement, to public activism in support of labor laws, economic equality, and environmental concerns, the past years have witnessed ample protests in the United States, Southeast Asia, and Latin America. Yet, the true epidemiological impact of gathering in support of social justice has seldom been analyzed (4, 5). In the unique Colombian example, a recent study (pre-print) tracked the effects on transmission rates of COVID-19 in five highly populated cities. It showed that of these, Cali and Bucaramanga experienced the closest correlations between increase in disease spread and rising social fervor (6).

Cali is the capital of the Valle del Cauca (VAC) department of Colombia. It is considered one of the country’s largest cities with approximately 2,227,642 inhabitants. Cali is also the city with the highest accumulated number of natural infections and deaths by SARS-CoV-2 in VAC as of 15 November 2021: 285,199 cases and 7,468 deaths, with a fatality rate of 2.62%^4^ and a seroprevalence of 30% (CI: 27--33%).^5^ Throughout the course of the third pandemic wave in 2021, Cali, along with other Colombian cities witnessed a number of protests and anti-government demonstrations that led to a major state of social unrest. However, Cali, the capital city of Valle del Cauca was home to the largest and most violent and unremitting demonstrations countrywide, thus being considered the epicenter of the protests. In fact, the continued and escalating state of civil arrest in Cali prompted the government to deploy the Armed Forces and block access to the city in an effort to contain protesters.

Taking into consideration this sustained increase in cases and deaths by SARS-CoV-2 during the protest period, as well as our still-precarious understanding of the epidemiological dynamics of the virus and variants of concern/of interest (VOC/VOIs), we planned a study evaluating the epidemiological and genomic behavior of SARS-CoV-2 from 4 April 2021 through 31 July 2021. To this end, we (i) evaluated circulating lineages, their phylogenomic relationships, and any clades circulating within the city and (ii) we deconstructed the mutational profile of each isolate, throughout the entire viral genome. Our results showed an undeniable relationship between these social gatherings and the increased incidence of COVID-19. Further, we identified four clusters (two of them associated with the Mu variant) with potential independent introductions in the city of Cali. Finally, we attempt to explain the decrease in number of circulating lineages (Mu variant dominance), during the time that the protests took place.

## Materials and Methods

### Epidemiological Data and Study Population

We analyzed case counts of Cali and Medellin from data deposited in the database of the Instituto Nacional de Salud, which can be accessed at: https://www.datos.gov.co/Salud-y-Protecci-n-Social/Casos-positivos-de-COVID-19-en-Colombia/gt2j-8ykr/data (accessed on 20 October 2021), as well as case counts of Bogota deposited in a public data source repository,^6^ from 4 April 2021 through 31 July 2021 (although this study focused on Cali, we wanted to include general estimates of similar populated cities for sake of comparison). This time period embraced a time of social unrest against the government dominated by riots, massive mobilizations, protests and walks that led to increased population circulation in the streets of different cities under deficient biosecurity conditions and lack of personal protective equipment. This database is available for public consultation and contains all variables used in our research, such as case notification date and deaths. Notification date corresponds to the date on which each suspect case was identified and reported to the Public Health System, and subsequently confirmed as positive. The metadata of cases, deaths and the estimated Rt value for each day and period evaluated in Cali city are described in the Supplementary Table 1.

Likewise, vaccination data was obtained from an open public data source,^7^ in which all vaccination events are recorded and its information used to determine the cumulative doses administered during a given period of time. Of note, accumulated doses included at this site, does not discriminate between first and/or second doses.

### Effective Reproduction Number Estimation

The effective reproduction number (Rt) was calculated using a Bayesian framework following the Cori et al. method (7) implemented in the Epyestim package^8^ with piecewise constant estimates on fixed arbitrary time intervals coinciding with the events (sub-periods previously described) of citizen protests occurring in all three main cities of Colombia where the largest riots occurred (Cali, Bogotá, and Medellin).

Effective reproduction number (Rt) was calculated based on the inference of infection events derived from SARS-CoV-2 positive cases designed as follows: New COVID-19 detected cases were smoothed using a LOWESS filter with a 21-day window and were subjected to two deconvolutions to infer the time series of infection events. The first deconvolution considers the delay from infection with SARS-CoV-2 to symptom presentation following a Gamma distribution with Alpha = 1.35 and scale = 3.77 (8). The second deconvolution considers the delay from symptom onset to detection by a test following a negative binomial distribution with Mu = 5.25 and Alpha = 1.57 (8).

### Sequencing and Bioinformatics Analysis

Samples for sequence analysis were obtained from patients attending the Dirección de Sanidad del Ejército. Nasopharyngeal swabs were placed in viral transport media LABG&M (Microgen Ltda., Colombia), and nucleic acid extraction was performed using the Quick-RNA Viral kit from ZYMO Research^®^ in a Hamilton Microlab Prep extraction platform, or Biomek i5 Nucleic Acid Extraction platform of Beckman Coulter, as well as b-Aid Virus RNA Extraction kit in a Lab-Aid 824s Nucleic Acid Extraction System of ZEESAN Biotech Co., following manufacturer’s recommendations. For the eluted RNA, SARS-CoV-2 detection was carried out by reverse transcription and multiplex amplification by Real-Time PCR (qPCR) using the VIASURE^®^ Kit (CerTest/Biotec) screening for ORF1ab and N gene targets (interpreting as positive those samples with a positive result for either of the two markers), or Allplex™ SARS-CoV-2 Assay of Seegene, in lyophilized format, with a mix of enzymes, primers, probes, buffer, dNTPs, stabilizers and an exogenous internal control by test. A positive test result was considered when both RdRp/ORFB1 and N genes were detected. Limit of detection (LOD) was 20 genome copies per reaction. Samples with a positive result and a Ct value <29 were stored at −20°C. Available samples were then grouped by epidemiological week and randomly selected for genomic sequencing. A total of 124 sequences were obtained from 4 April 2021 through 31 July 2021.

Whole genome sequencing of SARS-CoV-2 was performed using Oxford Nanopore’s MinION platform, using the MinKNOW application (v1.5.5) according to an established protocol.^9^ Bioinformatics analysis was performed as described in the ARTIC bioinformatics pipeline.^10^ Once the assemblies were obtained, typing was performed based on the Pangolin COVID-19 Lineage Assigner (Phylogenetic Assignment of Named Global Outbreak LINeages). The mutation search was performed by means of Clade assignment, mutation calling, and sequence quality checks NextClade v 1.5.4.^11^ A total of 228 sequences from Cali were herein obtained and analyzed from 4 April 2021 to 31 July 2021 (124 sequences sequenced in this study, and 104 sequences publicly available in the Global Initiative on Sharing All Influenza Data (GISAID) database in the same time period and the same location). For the moment of the data collection there were not genomes reported from Cali for the date 4 April 2021. Further information of the dates and genomes included in the analysis are included in the Supplementary Table 2. The abundance of SARS-CoV-2 variants over time was calculated from the complete Cali city genomes database (*n* = 228). The results were represented using the R software (9).

### Phylogenomic and Mutational Analysis

A dataset with 5,283 sequences was established with the aim of comparing the phylogenomic relationships of SARS-CoV-2 circulating in Cali (Colombia), and to infer the potential introduction dates for the most recent common ancestor (tMRCA). This data set included: (i) 228 sequences from Cali; (ii) 3,270 sequences from other areas of Colombia, including 170 from VAC, the greater geographical area which includes Cali (its capital city) (all the genomes available until the last date of our analysis, 31 July 2021); and 1,785 reference genomes representing the diversity of SARS-CoV-2 lineages available in the NextClade tool. All publicly available genomes were downloaded from the GISAID database (10). The new set of genomes was uploaded with the numbers registered in the Supplementary Table 2.

The complete dataset was aligned using MAFFT v7.40755 (11) with FFT-NS-2 algorithm and other parameters by default. The 5′- and 3′-untranslated regions were manually trimmed in Uniprot UGENE software v39.0^12^ considering the ORFs described for the reference strain Wuhan-1 (NC_045512.2). A maximum likelihood (ML) tree was then built in IQtree2 v.1.6.1 (12), using the best substitution model, default heuristic search options, and ultrafast bootstrapping with 1,000 replicates and other parameters by default as was previously described (13). A time-scaled ML phylogeny was then constructed in TreeTime (14) using the conditions: collection date as constraint and other parameters previously reported (15). All trees obtained were graphically represented in the Interactive Tree Of Life online tool (16).

Single-nucleotide polymorphism (SNP) analysis was performed by comparing the 228 genomes from Cali downloaded from the GISAID database (ranged from 4 April 2021 to 31 July 2021) with the reference genome from Wuhan, China (hCoV-19/Wuhan/Hu-1/2019, GenBank accession number: NC_045512.2) using the NextClade tool v 1.5.4 (see text footnote 11) (10), and the UGENE v.33.0 software (17). Additionally, the SNPs over time were evaluated (since 4 April 2021 to 31 July 2021).

### Statistical Analysis

A descriptive analysis of the sociodemographic variables collated in the databases was performed. The quantitative variables were summarized in terms of means or medians and standard deviation or interquartile range, based on their distribution. Qualitative variables were summarized as frequencies and proportions. Statistical analyses were carried out using R software. For continuous values, normality hypotheses were evaluated using the Kolmogorov–Smirnov test. All tests of significance, parametric or non-parametric tests, were two-tailed, and *p*-values < 0.05 were considered statistically significant.

## Results

### Epidemiology of Severe Acute Respiratory Syndrome Coronavirus 2 in Cali City

**FIGURE 1:**
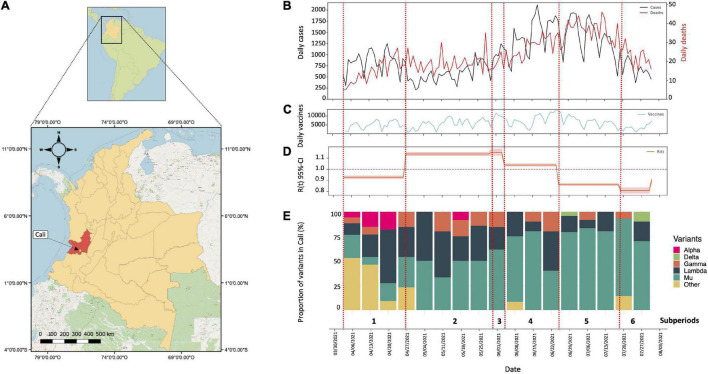
Genomic surveillance and epidemic model of SARS-CoV-2 in Cali from 4 April 2021 to 31 July 2021. **(A)** Geographical location of the department of Valle del Cauca and its capital Cali. The map was constructed using the QGIS tools (QGIS Geographic Information System, Open-Source Geospatial) version 3.20.3-Odense. Foundation Project (http://qgis.osgeo.org). **(B)** Number of cases (black line) and deaths (red line) for SARS-CoV-2 reported daily in Cali from 4 April 2021 to 31 July 2021. **(C)** Number of vaccines applied per day in Cali from 4 April 2021 to 31 July 2021. **(D)** Value Rt calculated from 4 April 2021 to 31 July 2021. **(E)** Distribution of SARS-CoV-2 variants in Cali, Colombia. Proportion of VOC/VOI and other variants in Cali, Colombia from 4 April 2021 to 31 July 2021.

We analyzed SARS-CoV-2 cases reported from 4 April 2021 through 31 July 2021, in Cali, Colombia (Figure 1A). A total of 115,167 positive cases and 2,795 deaths were reported during the period of active protests. On June 17, the city reached its COVID-19 incidence peak: with a total 2,117 confirmed cases (7 weeks after onset of protests) (Figure 1B). The highest number of virus-related deaths in this period occurred on July 12th, totaling 46 (11 weeks after protests began) (Figure 1B). Of the total number of cases reported during our study interval, 53% corresponded to female patients and 47% to male patients. The median age was 38 years (IQR: 27–52 years). Within the deceased group (*n* = 2,795), a higher proportion of male than female deaths (59%) was noted with a median age of 68 years (IQR: 58–79 years). The daily incidence was consistent with following mortality events (as shown in Figure 1B). It is important to highlight that throughout the time period studied, 720,325 total vaccine doses were dispensed to the population of Cali, corresponding to first and second doses (Figure 1C).

### Effective Reproductive Number (Rt) and Infection Dynamics

Based on information released by the government and media in relation to mass mobilizations and protests with highest popular circulation in the streets, we defined six sub-periods within our study-time interval (red dashed lines in Figures 1B–E). The initial sub-period: between April 4 and April 27 (prior to the beginning of the protests in the city). A second period, between 28 April 2021 (when the national strike began) and 30 May 2021 (when the mass mobilizations halted). A temporary arrest in mobilizations due to government negotiations with protesters defined a third sub-period from May 31 to June 4. Subsequent reactivation of the protests between June 5 and June 25, marked the fourth sub-period. Later, a new “return to normal” took place between June 26 and July 19, marked by a re-establishment of dialogs between the government and citizens (fifth sub-period). However, due to an incapacity for agreement, massive demonstrations reignited in July 20 through July 31 setting the sixth sub-period. July 31 thus defines the ultimate date for this analysis.

**FIGURE 2:**
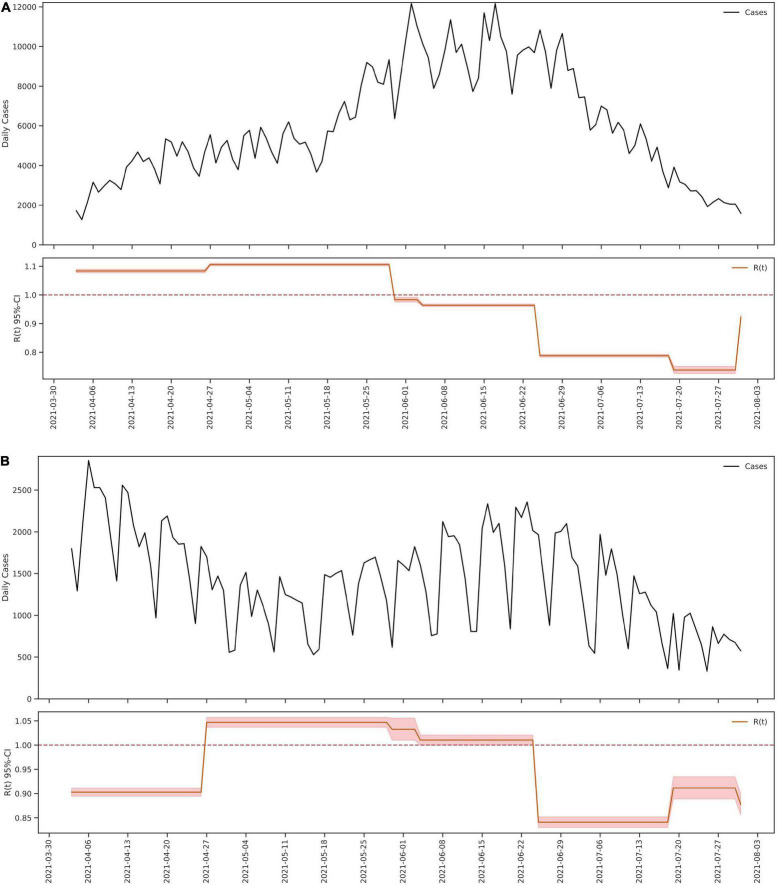
Epidemic model of SARS-CoV-2 in Bogota and Medellin from 4 April 2021 to 31 July 2021. The figure shows the number of cases for SARS-CoV-2 reported daily and the value Rt calculated from 4 April 2021 to 31 July 2021, in the cites of Bogota **(A)** and Medellin **(B)**.

Based on these sub periods, the analysis of the effective reproduction number (Rt) was calculated using a Bayesian framework following the Cori et al. method (7). This method allows understanding changes in the Rt value at discretized times such as those herein established. The Rt values were significantly different between the time periods evaluated (Kruskal–Wallis Chi-squared = 112.5, *p* < 0.05) in this study (Figure 1D). Prior to the beginning of the national strike in Colombia, the city of Cali had an Rt of 0.89 and short after the initiation of unrest this value steadily increased through the second and third sub periods, exceeding the threshold of 1 (1.12 and 1.13, respectively), being statistically higher for both cases (Kruskal–Wallis Chi-squared = 112.5, *p* < 0.05. Dunn–Bonferroni *post hoc* Test, *p* < 0.05). Likewise, during cessation of the strikes between June 26 and July 19, a decrease in the Rt value to 0.84 was noted, even lower than in the previous periods marked by massive mobilizations greater circulation of people in the streets (Kruskal–Wallis Chi-squared = 112.5, *p* < 0.05. Dunn–Bonferroni *post hoc* Test, *p* < 0.05) (Figure 1D). These results contrast with those observed in Bogotá (Figure 2A) and Medellin (Figure 2B) were the largest riots occurred, exhibiting just a slight increase in the Rt value (very close to 1), both prior to the beginning of the protests as in the period of largest mobilizations (Figure 2).

Based on the analysis of SARS-CoV-2 genome assemblies, available during the six evaluated sub-periods (4 April 2021 and 31 July 2021), we identified circulation of 16 SARS-CoV-2 lineages (with a lineage proportion of 50.44% lineage B.1.621, 24.12% C.37, 7.46% P.1, 4.39% B.1.1.348, 3.95% B.1.621.1, 2.63% B.1.1.7, 1.32% B.1, 0.88% lineages A.2.5, B.1.1, B.1.625, and B.1.623; and with a proportion of 0.44% the lineages AY.20, AY.26, B.1.1.487, C.37.1, and P.1.10), with the greatest diversity of lineages (9 lineages circulating) being represented in the first sub-period (between 4 April 04 2021 and 27 April 2021) when protests had yet not started. At this time, approximately 50% of the variants were classified as VOCs/VOIs with the remaining 50% classified as B.1.625, B.1.1, and some of its descendant lineages (e.g., B.1.1.348 and B.1.1.487) (Figure 1E). Throughout the second sub-period (28 April 2021), we noted a decrease in the number of circulating lineages, as well as an increase in prevalence of VOCs and VOIs, specifically, of Lambda, Gamma, and Mu variants. Interestingly we observed a significant increase in the number of cases associated to the Mu variant (Figure 1E); particularly 2 weeks after initiation of the national strike (second sub period), a trend that was maintained during the remaining periods evaluated.

### Phylogenomic Relationships and Potential Introduction Dates

**FIGURE 3:**
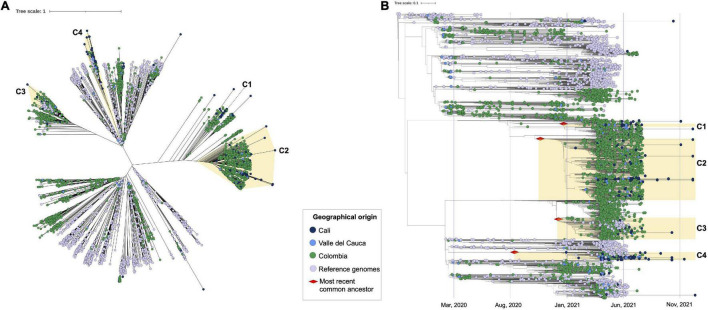
Phylogenomic relationships of evaluated SARS-CoV-2 genomes in the global context, identification of the most recent common ancestor (tMRCA). **(A)** Phylogenomic relationships between the 5,283 genome sequences analyzed, which included 228 sequences from Cali (blue dots) 3,270 sequences from other areas of Colombia [green dots, including Valle del Cauca (magenta dots)]; and 1,785 reference genomes (purple dots). The clusters consisting of 179 of the 228 genomes from Cali are highlighted in light yellow (C1–C4). **(B)** Phylogenomic relationships between the 5,283 genome sequences analyzed with the identification of the most recent common ancestor (tMRCA). The red diamond represents the putative introduction date per each cluster.

The alignment obtained from the complete dataset (*n* = 5,823) including 228 genome sequences available from Cali, and 170 from the greater Valle del Cauca (labeled set as VAC) was used to infer a ML phylogeny in IQtree2. Tree topology revealed that the SARS-CoV-2 genome sequences from Cali mostly grouped (*n* = 179) into four main clusters, with the remaining 49 genomes being heterogeneously distributed (Figures 3A,B). Two of these clusters (C1 and C2, where the Mu variant predominated) were closely related. In the case of C1, this included 57 sequences mostly from Colombian genomes, with 21 sequences from VAC (9 from Cali and 12 from elsewhere in the department). The second cluster (C2), whose predominant variant was Gamma, comprised 1,140 sequences, including 109 genomes from VAC (54 from Cali and 55 from other regions of VAC) plus six reference genomes mostly from United States (*n* = 7) and one from England. The third cluster (C3), whose predominant variant was Lambda, contained 395 genomes comprising 18 from VAC (6 from Cali and 12 from other regions of VAC), 61 reference sequences mostly from United States, and only three genomes from South America (two from Brazil and one from Uruguay), with the reminder genomes deposited from European countries. Lastly, the fourth cluster (C4) embraced 147 sequences with 61 from VAC (37 from Cali and 24 from its department), that were closely related with 31 reference genomes mostly from United States (*n* = 35) and 2 from Peru. All genomes not mentioned during the description of the clusters belonged to different departments of Colombia, with a predominant profile from Antioquia (Northwestern Colombia).

In addition, tMRCA for the VOCs/VOIs identified in the 228 genomes from Cali, was inferred using TreeTime (Figure 3B). The results showed two potential introduction events for the Mu variant (C1 and C2), the first on January 6 2021 (95% CI = December 7 2020 to February 10 2021) and the second on November 3 2020 (95% CI = September 22 2020 to November 24 2020). These genomes were closely related to other Colombian genomes suggesting that these two independent introductions occurred most probably from other departments. On the other hand, the putative introduction for Gamma variant (C3) was estimated in December 27 2020 (95% CI = December 12 2020 to January 13 2021) and for the Lambda variant (C4) in August 24 2020 (95% CI = January 26 2020 to November 24 2020); with genomes in close relationship to those from other countries (United States, Brazil, Uruguay, Europe, and Peru), suggesting independent introductions to Cali from those countries.

### Mutational Analysis

**FIGURE 4:**
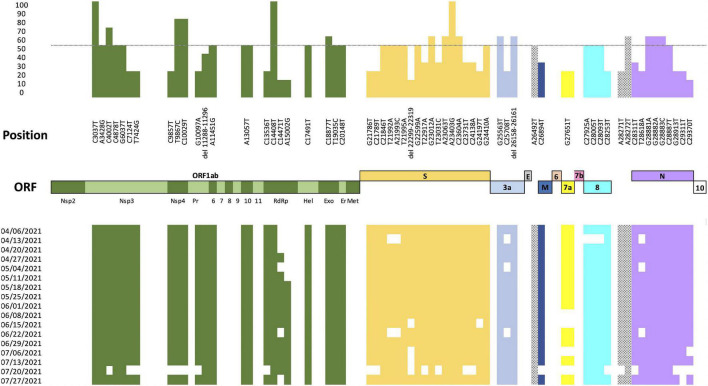
Nucleotide diversity of the Cali genomes. Analysis of nucleotide diversity by comparing the 228 Cali SARS-CoV-2 genomes and the Wuhan reference sequence (hCoV-19/Wuhan/Hu-1/2019), GenBank accession number: NC_045512.2 over time. The upper panel represents in the *X*-axis the position of each mutation within the SARS-CoV-2 genome and in the *Y*-axis the percentage of genomes with the mutation. The lower panel shows the presence (represented with color) or absence (represented without color) of each substitution found in the 228 Cali genomes analyzed between April 4 2021 and July 31 2021.

Fifty-eight SNPs and three deletions over more than 10% of SARS-CoV-2 genomes from Cali (*N* = 228) were identified (Figure 4). Thirty-three of the substitutions (57%) were identified as non-synonymous substitutions, most of them identified in VOC as Alpha and Gamma and in VOI as Lambda and Mu variants. Additionally, the analysis of these mutations through time (April 4 2021 and July 31 2021) revealed that the substitution A15002G (ORF1b) was introduced on May 18 2021 and was maintained during the analyzed period (Figure 4).

## Discussion

According to the Armed Conflict Location & Event Data Project (ACLED), more than 100 countries have been witnessed to internal civil disorder. Of these, India, Israel, and Mexico have been most afflicted by such events in relation to the development of the COVID-19 pandemic within their borders (18). Extensive domestic and international debates of “right-and wrong” have been encouraged by media outlets documenting these country-specific social issues. Yet, conversations concerning the true impact of civil unrest on the epidemiology of COVID-19 remain poorly discussed. In this article, we investigated the epidemiological and genomic behavior of SARS-CoV-2 as it was forcibly integrated into the population by social turmoil in Cali, Colombia. We chose to examine this particular city for several reasons. Primarily because Cali was the third city, after Bogotá and Medellin with the highest accumulated number of natural infections and deaths by SARS-CoV-2 during the study period (April 4 2021 to July 31 2021). In addition, Cali was the city where the majority and most severe acts of vandalism and convulsed demonstrations occurred, leading to sustained chaos while anticipating a potential rebound for virus transmission.

Among the analyses central to the conclusions of this article, we highlight the Rt values in Cali, Colombia, gathered from the periods during, and prior-to/post the most significant period of demonstrations (April 28 2021 to June 25 2021) (Figure 1D). The Rt value being greater than one during this period, as opposed to less than one at other times. We can make an association between the assembly of large groups, and the heightened incidence of COVID-19 cases with the support of these Rt values and with the evidence of a peak in daily cases documented 2 weeks after the largest protests took place (Figure 1B). However, it is important to note that other events, such as the lifting of the restrictions, which took place in a moderate and staggered manner and the Easter holidays (March 28–April 4) could have had effect in the increase of Rt value.

Our findings are aligned with the findings by Moreno-Montoya et al. (6) and Valentine et al. (5), which describe a positive growth in the occurrence of COVID-19 cases after the protests; but were contrary to those of Neyman et al. (4), which indicated that each individual protestor did not significantly contribute to the COVID-19 case rate in affected countries. Nor did our findings align with previous studies by Bui et al. (19)—which did not reveal a significant relationship between protests and COVID-19 hospitalization rates within California counties. Some possible explanations for these disparities may include a greater neglect of social distancing guidelines experienced during the dissents in Colombia, failure to use personal protective equipment, inadequate ventilation among areas shared by the large groups, the lower vaccination rate within this city: (720,325), which corresponds to 32% of the total population (Figure 1C) and the high rate of transmissibility of SARS-CoV-2, which has been associated with the ability of this virus to replicate extensively in bronchial and alveolar epithelia, the high “silent” presymptomatic transmission (20) and the reproductive number R0 (average number of secondary cases generated per typical infectious case), which for SARS-CoV-2 present a median point estimate of 3.1 (21). Additionally, we consider that Mother’s Day celebration (May 9 2021), which coincided with the period of protests could have contributed to increased SARS-CoV-2 transmission due to greater family gatherings at that time, in a similar fashion to what has been reported for holiday gatherings in the United States (22). This could very well have augmented the statistic describing COVID-19 spread in the whole of the city during the evaluated period.

Our analysis of 228 SARS-CoV-2 genomes, revealed 16 independent lineages of the virus circulating in Cali at one time or another during the period of heaviest social demonstrations. These included three VOCs Alpha, Delta, and Gamma, and two VOIs Mu and Lambda (Figure 1E). These findings are significant given the increased infectivity and transmission potential, severe clinical outcomes, and evasion properties of protective antibodies from previous antigen exposure, which have all become defining signatures of these variants (22–25).

Of the 16 lineages, only 9 had been circulating in the initial weeks of the protests. As the months progressed, we saw a decrease in the number of lineages coupled to an increase in the number of cases attributed to the highly infections Mu variant (Figure 1E). Many infectious diseases propagate in a population when migration of masses of non-immune individuals enter the physical domain of people amongst whom herd immunity is attaining its first grip. Such is the case in support of our findings: the arrival of large numbers of people to Cali, during this period of turmoil (either those supporting protests against President Iván Duque, as in the case of *Minga* indigenous individuals from Cauca department –Southern VAC–, or those sent to control protesters as was seen with the arrival of military personnel from exterior municipalities) aided the profusion of SARS-CoV-2 throughout the city.

Sustained movements of people into and out of the city likely further favored the dispersion of the Mu variant in Cali and in other regions of Colombia. The Mu variant is thought to have been introduced to the city at two independent periods, preceding the protests, one on November 3 2020, and later on January 6 2021 (Figure 3B). It may be further inferred that these independent occasions primed the predominance of the Mu strain over all other lineages of SARS-CoV-2 and the 55% increase in the number of reported cases during the period in which the protests took place (Figure 1E).

In January 2021, the Mu variant (completely defined as B.1.621/B.1.621.1), was first identified in Colombia (26). To date, it has been described (with its sub-lineage B.1.621.1) in more than 30 countries including the United States, Spain, Mexico, Hong Kong, Netherlands, and Denmark (27, 28). Its remarkable efficiency of transmission in a population might be explained by an excellent immune-evasion capacity of 0.38 (CrI: 0.32–0.43), as compared against the Gamma variant: 0.30 (CrI: 0.24–0.33) (28). Further, the Mu variant evades both antibodies produced by natural infection, as well as those by induced immunization *via* vaccine—Mu variant being 12.4 times more resistant to neutralization by convalescent sera in the former case (28), and 7.6-fold more resistant to sera obtained from BNT162b2-vaccinated individuals, in the latter (28–30). It is thus important to note that the transmission behavior and potential of COVID-19 examined during these protests should be primarily, if not principally, attributed to the Mu variant.

Previous studies have speculated that the un-anticipated dominance of the Mu strain over other VOCs and VOIs, proceeds from its unique capacity to evade the immune system (26). Despite a subpar transmission index (1.34; CrI:1.22–1.43), as compared with the Gamma variant (1.86; CrI:1.63–1.90) (28), it seems that the aforementioned serum antibody resistance confers evolutionary advantage in populations among which other VOCs circulate still. The Gamma variant was introduced to Cali at about the same time as was the Mu variant (Figure 3B) yet viral spread in Cali was superseded by Mu during the full course of the period of fervent demonstrations. Further advanced studies are needed to make more poignant claims on possible correlations between the molecular makeup of Mu, and its persistence in a population. In this context, we emphasize that it is indispensable to follow through with SARS-CoV-2 genomic surveillance and vaccination programs in Colombia. This is reinforced by the recent emergence of the omicron lineage—which yet remains a mystery as it quickly continues to propagate worldwide.

This is the first such report of the epidemiological and genomic behavior of SARS-CoV-2 in the framework of Colombia’s recent turbulent period of popular protests against the government. The results of our study demonstrate that these large group assemblies in Cali, together with the mass movements into and out of the city during the summer of 2021 favored the spread of SARS-CoV-2 (specifically of the Mu variant). The heightened spread of COVID-19, increased hospitalizations in Cali and consequently saturated the city’s intensive care units (95% saturation). At present, 48.1% of the Colombian population is vaccinated and VOCs like Alpha, Gamma, and Delta, along with VOIs like Mu and Lambda co-circulate in our country. We consider therefore, that persistent dialogue between the public health directory in the government, and the Colombian people is imperative in order to halt further transmission chains.

Finally, we note some limitations to our study: we recognize a very probable under-reporting of the true number of COVID-19 cases in Cali during the period of interest. The abstinence of these cases from the true percentage of people infected during the summer of 2021 in Cali may be attributed to asymptomatic carriers of the virus. Second, we remain in the dark about many phenotypic characteristics of the SARS-CoV-2 strains assessed as new strains appear in the global circuit as the character of those evolutionarily preceding them is still being elucidated. Thus, we may be missing key points about virulence, transmission, and immune evasion which would have better explained the dynamics of viral spread in Cali during the period in question. Third, with a rather low number of SARS-CoV-2 genomes sequenced, we were only allowed to have a snapshot of the greater picture. This may have been a sample which coincidentally drew one conclusion in place of another. Finally, the environment of Cali may have favored one dispersion outcome over others possibly driven by the nature of the variants, had they been distributed in a different setting. The scenario presented here portrays a clear example on the significance of the interplay between viruses, environment and their interactions with host populations, particularly in context of complex human interfaces as seen during social conflict.

## Data Availability Statement

The datasets presented in this study can be found in online repositories. The names of the repository/repositories and accession number(s) can be found in the article/Supplementary Material.

## Ethics Statement

The studies involving human participants were reviewed and approved by the Universidad del Rosario Ethics Committee. The patients/participants provided their written informed consent to participate in this study.

## Author Contributions

LP conceived and designed the study, analyzed and interpreted the data, and prepared the manuscript. SC and EG-A carried out the bioinformatic analysis. MM carried out the phylogenomic analysis. NB, AR, NL, JP, CC-C, MD, CM, CO, MS, and AP-M critically revised the manuscript and made important suggestions. JR conceived and designed the study, and prepared and revised the manuscript. All authors have reviewed and approved the manuscript.

## Conflict of Interest

The authors declare that the research was conducted in the absence of any commercial or financial relationships that could be construed as a potential conflict of interest.

## Publisher’s Note

All claims expressed in this article are solely those of the authors and do not necessarily represent those of their affiliated organizations, or those of the publisher, the editors and the reviewers. Any product that may be evaluated in this article, or claim that may be made by its manufacturer, is not guaranteed or endorsed by the publisher.

## Footnotes

1
https://coronavirus.jhu.edu/map.html

2
https://www.ins.gov.co/Noticias/Paginas/Coronavirus.aspx

3
https://www.minsalud.gov.co/salud/publica/Vacunacion/Paginas/Vacunacion-covid-19.aspx

4
https://www.ins.gov.co/Noticias/Paginas/coronavirus-filtro.aspx

5
https://www.ins.gov.co/estudio-nacional-de-seroprevalencia/reporte.html#curso

6
https://datosabiertos.bogota.gov.co/dataset/44eacdb7-a535-45ed-be03-16dbbea6f6da?_external=True

7
https://datos.cali.gov.co/dataset/registros-de-vacunas-para-covid-aplicadas

8
https://github.com/lo-hfk/epyestim

9
https://artic.network/ncov-2019

10
https://artic.network/ncov-2019/ncov2019-bioinformatics-sop.html

11
https://clades.nextstrain.org/

12
http://ugene.net/
